# Development and Evaluation of a New qPCR Assay for the Detection of *Mycoplasma* in Cell Cultures

**DOI:** 10.3390/cimb45080435

**Published:** 2023-08-18

**Authors:** José A. Carrillo-Ávila, Amanda de la Fuente, Rocío Aguilar-Quesada, Gertrudis Ligero, Juan Manuel del Río-Ortiz, Purificación Catalina

**Affiliations:** Andalusian Public Health System Biobank, Coordinating Node, Av. del Conocimiento, S/N, 18016 Granada, Spain; amandadlafuente@gmail.com (A.d.l.F.); rocio.aguilar.quesada@juntadeandalucia.es (R.A.-Q.); gertrudis.ligero@juntadeandalucia.es (G.L.); juanm.rio@juntadeandalucia.es (J.M.d.R.-O.); purificacion.catalina@juntadeandalucia.es (P.C.)

**Keywords:** cell culture, *Mycoplasma*, PCR, 16S rDNA, cell line contamination, biobank

## Abstract

In recent years, cell culture has become an important tool not only in research laboratories, but also in diagnostic and biotechnological development laboratories. *Mycoplasma* contamination is present in up to 35% of cell cultures used in research and in cell therapies. This fact represents a significant problem since such contamination can cause disastrous effects on eukaryotic cells by altering their cellular parameters, which, in turn, can lead to unreliable experimental results. For this reason, it is mandatory to carry out continuous testing for the presence of *Mycoplasma* in cell culture and the development of appropriate methodologies for this purpose. An ideal detection methodology should be fast, sensitive, and reliable. In this study, we propose an alternative detection method based on real-time PCR in conjunction with a novel combination of primers and probes that have been improved to increase their efficiency. The new PCR method demonstrates 100% sensitivity and specificity results in the detection of common *Mycoplasma* species that contaminate cell cultures. Whilst 11 of 45 tested supernatants were positive for *Mycoplasma* (24.4%) using the new PCR method (corresponding to 5 of the 14 lines tested (35.71%)), only 10 of 45 supernatants showed positive results with the commercial Venor^®^GeM qEP and Plasmotest^®^ kit. In addition, the new PCR method exhibits a high capacity to detect less-frequent *Mycoplasma* species, such as those related to the *M. mycoides* cluster. The use of an alternative *Mycoplasma*-detection method in cell culture labs can guarantee the detection of *Mycoplasma* contamination, especially in cases when dubious results are recorded.

## 1. Introduction

Animal cell cultures have become a basic, frequent, and fundamental tool not only in research laboratories, but also in diagnostic and biotechnological development laboratories [1]. In this sense, animal cell lines are used in in vitro models to investigate new therapeutic approaches against different diseases [2,3]. For this reason, it is essential that cell cultures are kept aseptic. Although different microorganisms (bacterial, fungi, yeast, or viruses) are capable of contaminating cell cultures [4], *Mollicutes* (or interchangeably *Mycoplasma*) are the fundamental cause of contamination. Despite the availability of sophisticated technology and specialisations used in manipulating cell cultures, biological contamination primarily due to *Mycoplasma* contamination remains a significant challenge.

*Mycoplasma* contamination of cell cultures was first described 50 years ago [5], and it is claimed that approximately 5–35% of current cell cultures are contaminated [6]. The *Mycoplasma* genus comprises microorganisms with distinctive characteristics, including high resistance to antibiotics [7], reduced metabolism, the lack of a rigid cell wall, a small size (0.3–0.8 μm), and high flexibility, so they are able to pass through common antibacterial filters (0.45 μm) [8]. These microorganisms grow slowly, even if they are in optimal conditions, and also have a long latency phase where they persist for a long time without being obvious. This is due to its limited biosynthetic capabilities and the obligatory dependence on eukaryotic cells. During this time, these microorganisms obtain nutrients both from the culture medium and from the metabolism of eukaryotic cells [7].

*Mycoplasma* contamination is a significant problem in animal cell lines. The impact of *Mycoplasma* on different cell types varies. Furthermore, the degree of cytopathogenesis also varies among different *Mycoplasma* species, something that, on occasion, goes unnoticed, which is the reason why respective contamination is not readily detected [8,9]. However, *Mycoplasmas* can extensively affect the host’s DNA, RNA, and protein metabolism, impact intracellular amino acid and available ATP levels, modify cellular surface antigens, and provoke the fragmentation of DNA and other significant chromosomal alterations [9]. This seriously impacts cell viability, gene expression, morphology, metabolism, and the growth rate of infected cells [10,11]. Consequently, scientific results based on contaminated cell cultures are unreliable and pose a significant obstacle for the pharmaceutical industry and its work on cell therapy [12,13].

The primary sources of *Mycoplasma* contamination are foetal bovine serum or other contaminated laboratory reagents, incorrect sample handling by laboratory personnel, and cross-contamination with other contaminated laboratory lines, for example, as a result of an exchange of cell lines between different laboratory and research groups. It is worth highlighting the importance of cell manipulation by laboratory personnel, which means that there is transfer of *Mycoplasma* through laboratory equipment, through culture media, sera, and reagents, in incubators, the contamination of liquid N_2_ in tanks in which the contaminated cells are stored, and transmission via airborne particles and aerosols. In this way, *M. orale*, *M. fermentans*, and *M. horminis* account for half of all infections in cell cultures and are species found physiologically in the human oropharyngeal tract [9].

Different methodologies have been developed to detect *Mycoplasma* in cell cultures. The World Health Organization has proposed using harmonising assays for *Mycoplasma* DNA detection [14]. At present, numerous methods with different sensitivities and specificities regarding *Mycoplasma* testing are available, including microbiological culture, direct DNA staining (Hoechst), colorimetric detection assays, and Nucleic Acid Amplification Techniques (NAT assays) [15]. Although the microbiological culture method has been considered the ‘gold standard’ for detecting viable *Mycoplasma*, this specific testing method is time-consuming (a minimum of 28 days) [16,17]. The most widely used and sensitive tests are NAT assays in their different variations, i.e., quantitative, semiquantitative, or qualitative assays [18,19,20].

Of the more than 200 known species of *Mycoplasma*, a limited number of species (namely, *M. arginini*, *M. fermentans*, *M. hominis*, *M. hyorhinis*, *M. orale*, *M. pneumoniae*, *Acholeplasma laidlawii*, *Spiroplasma citri*, and *Ureaplasma*) is responsible for more than 90% of instances of cell culture contamination [7,21]. However, only a few NAT-based kits are currently available [22], some of which are hard to obtain in Spain. Even still, some kits use identical protocols and only guarantee the detection of the most common species of *Mycoplasma* or fail to detect some common species bellow 10 CFU/mL [22]. Notwithstanding this, the need to design an alternative PCR for the specific, wide-ranging, and simultaneous detection of all common *Mollicutes* has been demonstrated since it has been observed that, in some cases, the primers that are used in some NAT-based kits lack specificity, whilst, in others, the sensitivity of the assay is not uniform across the analysed species [22,23,24,25,26].

The Andalusian Public Health System Biobank (BBSSPA), as a fundamental part of the National Bank of Cell Lines (BNLC), is in charge of depositing cell lines for their assignment to research projects that may require them. This deposit process includes traceability control through Short Tandem Repeat (STR) analysis, *Mycoplasma* contamination control, and karyotype and any other genetic analysis necessary to verify the suitability of the deposited line. Among these control processes, testing for the absence of *Mycoplasma* is essential to guarantee the absence of altered gene expression and cellular metabolism that could compromise future experiments. In our experience, we have observed the detection of sometimes uncertain or confusing results. The importance of successfully detecting *Mycoplasma* contamination in cell cultures, in conjunction with the current lack of an infallible commercial methodology that can be used to detect *Mycoplasma* contamination, led us to develop an alternative, highly sensitive, real-time PCR assay detection method that can be used in our laboratory and in the scientific community to assure the correct analysis of *Mycoplasma* contamination. The developed method is based on the selective amplification of a DNA fragment using a new and original set of universal and degenerate primers targeting the 16S rDNA region from a large selection of the *Mycoplasma* species genomes.

## 2. Materials and Methods

### 2.1. Supernatant Samples

A total of 14 cell lines were received in the BBSSPA between February 2018 and December 2019. These cell lines were cultivated at different passages, and a total of 45 supernatants from 14 cell lines were tested for *Mycoplasma* detection (Table 1). Each supernatant sample (1 mL) was recovered and stored at −80 °C until the time of testing using NAT assays.

### 2.2. Samples for Cell Culture Screening

The samples were processed according to Venor^®^GeM qEP guidelines for analysis using NAT assays. The *Mycoplasma*-detection process involved a simplified DNA isolation protocol that did not require an additional DNA isolation kit. For each sample, 500 μL of supernatant was transferred to a 1.5 mL tube and was incubated at 95 °C for 10 min. After a brief period of centrifugation, 2 μL of each sample was used for qPCR detection.

### 2.3. Standard DNA

A *M. fermentans* DNA standard (Promocell, Heidelberg, Germany, Cat. No. PK-CA91-0117) containing the strain NCTC 10117 was used for qPCR optimisation. The DNA was supplied at 1 × 10^6^ copies/μL.

A collection of *Mycoplasma* Sensitivity Standards (Minerva Biolabs, Heidelberg, Germany, Cat. No. 102-0002) containing 10 CFU/mL was also used to assess the sensitivity and specificity of the qPCR assay. The standards contain inactive *Mycoplasma* DNA of 9 species: *A. laidlawii*, *M. arginini*, *M. fermentans*, *M. gallisepticum*, *M. hyorhinis*, *M. orale*, *M. pneumoniae*, *M. synoviae*, and *S. citri*, as well as a negative control sample. The lyophilised material was reconstituted using 1 mL of Essential 8^TM^ DMEM/F12(Ham) (1:1) culture medium (ThermoFisher Scientific, Waltham, MA, USA, Cat. No. A1516901). After 5 min of incubation at room temperature, the samples were vortexed for 10 s. Finally, a Venor^®^GeM Sample Preparation Kit (Minerva Biolabs, Heidelberg, Germany, Cat. No. 56-1200) was used to purify the *Mycoplasma* Standards. Then, 200 µL of the standards was treated and isolated as a sample following the manufacturer’s recommendations. The sample was subsequently resuspended in 60 µL of resuspension buffer.

### 2.4. The Design of Primers and Probes

The study and alignment of the 16S rDNA regions of different *Mycoplasma* species were performed, including species known to commonly contaminate cell cultures and unusual species [7,8,10]. The targeted species included *M. pneumoniae* (NR_041751.1), *M. gallisepticum* (NR_104952), *M. bovis* (NR_102850), *M. fermentans* (NR_044666), *M. synoviae* (NR_044811), *M. hyorhinis* (NR_041845), *M. orale* (AY796060), *M. salivarium* (NR_041745), *M. hominis* (NR_041881), *M. arginini* (LC158832), *Acholeplasma laidlawii* (NR_07444.8), *Spiroplasma citri* (NR_036849), *M. bovis* (NR_102850.2), *M. penetrans* (NR_118664.1), *M. urealyticum* (NR_041710.1), *M. buccale* (AB680682.1), *M. felis* (U09787.1), *M. dispar* (AF412979.1), *M. microti* (AF212859.1), *M. conjunctivae* (U44770.1), *M. canis* (AF340023.1), *M. hyopneumoniae* (GU227406.1), and *M. capricolum* (DQ157864.1). In addition to these species, the 16S rDNA regions from other bacteria, including *Escherichia coli* (NR_024570.1), *Chlamydia pneumoniae* (NR_026527.1), *Legionella pneumophila* (NR_041742.1), and *Streptococcus pneumoniae* (NR_028665.1), were considered, thereby avoiding cross-reactions. A sequence alignment of the 16S rDNA regions was performed using the Unipro Ugene v38.0 software.

Due to the high number of *Mycoplasma* species that must be detected and the significant degree of diversity in the sequences across the different species examined for this study, only limited areas of the 16S rDNA gene can be used in the design of primers and probes that are common to most *Mycoplasma* species. Therefore, as proposed by Uphoff and Drexler [8], an equimolecular mixture of the six proposed forward primers was used as a forward primer, except where two of them were simplified into a single primer in anticipation of a degenerate position; specifically, we used the primer CGCCTGAGTAGTACGTWCGC instead of CGCCTGAGTAGTACGTTCGC and CGCCTGAGTAGTACGTACGC, as well as the primer TGCCTGRGTAGTACATTCGC instead of TGCCTGAGTAGTACATTCGC and TGCCTGGGTAGTACATTCGC, as proposed by Uphoff and Drexler [8]. Furthermore, we designed a new reverse primer to amplify a fragment of approximately 256 bp in length. For qPCR detection, we selected a new ‘FAM’-labelled probe (Table 2) designed based on the small region conserved in all selected *Mycoplasma* species and included it between the forward and reverse oligos used. All of the primers and probes were supplied by Sigma-Aldrich.

**Figure 1:**
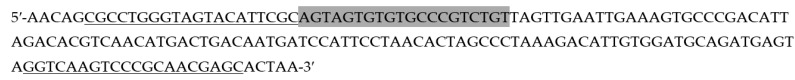
The synthetic DNA fragment used as an internal control. The locations of the primers for the PCR are underlined. The location of the internal probe is shaded.

A synthetic DNA fragment (169 bp) was designed as an internal amplification control. This synthetic amplification sequence was designed to be amplified using the same forward and reverse primers employed in *Mycoplasma* amplification but presenting a different internal sequence. A specific HEX label probe was used for the internal control, as depicted in Figure 1. This fragment was synthesised by chemical synthesis and cloned into a pMA-T vector by the GeneArt gene synthesis service (ThermoFisher Scientific, Waltham, MA, USA), providing us with a 2543 bp plasmid.

### 2.5. qPCR Conditions

The optimal qPCR conditions were established to guarantee the maximum sensitivity using serial dilutions of an *M. fermentans* DNA standard (1 × 10^5^ copies/μL to 1 copy/μL). Primers and probes described in the previous section were tested using a TaqMan Fast Advanced Master Mix (ThermoFisher Scientific, Waltham, MA, USA, Ref: A44260) in a QuanStudio-6 thermocycler (ThermoFisher Scientific, Waltham, MA, USA). Different final primer concentrations for *Mycoplasma* (250, 500, 750, and 1000 nM) were tested for the sep-up of the assay. In the same way, five probe concentrations for *Mycoplasma* and for the internal control (Myco-S1 Probe and Myco-IC-S1 Probe, respectively) (62.5, 93.75, 125, 187.5, and 250 nM) were tested. In addition, different dilutions of the internal control, between 1 × 10^−4^ and 1 × 10^−9^ copies/μL, were tested to guarantee correct detection without compromising the efficiency of the test.

Finally, after qPCR optimisation, each PCR reaction was prepared in a final volume of 20 μL, containing 10 μL TaqMan Fast Advanced Master Mix (2×), 1 μL of 10 μM primers (U&D-F (Myco U&D-F1, Myco U&D-F2, Myco U&D-F3, and Myco U&D-F4) and Myco-R5) (final concentration of 500 nM), 0.75 μL of 5 μM Myco-S1-Probe (final concentration of 187.5 nM), 0.375 μL of 5 μM Myco-IC-S1-Probe (final concentration of 93.75 nM), 1 μL of an internal control plasmid (1 × 10^−6^ ng/μL), and 4.875 μL of water. Finally, 2 μL of the sample was added. For the PCR program, a 20 s initial denaturation was used, followed by 45 cycles with 1 s at 95 °C and 20 s at 60 °C for annealing and extension, following the manufacturer’s recommendations. Fluorescent readings were performed for FAM and HEX fluorochromes.

### 2.6. qPCR Mycoplasma Detection with a Venor^®^GeM qEP Kit

A Venor^®^GeM qEP (Minerva Biolabs, Berlín, Germany, Ref: 11-9100) kit was used for *Mycoplasma* detection following the manufacturer’s instructions. The kit is designed to provide positive tests for *A. laidlawii*, *M. hyorhinis*, *M. fermentans*, *M. orale*, *M. synoviae*, *M. pneumoniae*, *M. arginini*, *M. gallisepticum*, *S. citri*, *M. arthritidis*, *M. genitalium*, *M. hominis*, *M. penetrans*, *M. salivarium*, and *Ureaplasma urealyticum*. Two microlitres of pre-treated supernatant was used in a 25 μL final PCR volume following the PCR program, as recommended by the manufacturer. Using a QuanStudio-6 Thermocycler, FAM and HEX wavelengths were employed for *Mycoplasma* detection and internal control detection, respectively.

### 2.7. Purification of Amplified Bands

The amplified fragments were separated in a 2% agarose gel at 60 V. Specific *Mycoplasma* bands (≈256 bp) were extracted from the agarose and purified with Buffer QG (Qiagen, Hilden, Germany, Ref: 19063) and a QIAquick PCR purification kit (Qiagen, Hilden, Germany, Ref: 28104) according to the manufacturer´s instructions. The amplicons were resuspended in 30 μL of Buffer EB and quantified (A_260_) using a Nanodrop EP spectrophotometer (ThermoFisher Scientific, Waltham, MA, USA).

### 2.8. DNA Sequencing

The purified 16S rDNA fragments were used in sequencing reactions with a BigDye Terminator v3.2 Cycle Sequencing kit (Applied Biosystems, Waltham, MA, USA, Ref: 4336917) using a Seq Studio Genetic Analyzer (Applied Biosystems, Waltham, MA, USA). The sequencing reactions were prepared in a final volume of 10 μL, with 20 ng of DNA, 3.2 pmol of the forward or reverse PCR primers (Table 2), and 4 μL of BigDye Terminator 3.1 Ready Reaction Mix. An equimolecular mix of Myco U&D-F1, F2, F3, and F4 was used as a forward primer, with Myco-R5 as a reverse primer. The sequencing reaction was performed using a thermocycler (Eppendorf EP gradient S, Hamburg, Germany) with an initial incubation step at 96 °C for 1′ and 25 cycles (96 °C for 10″, 50 °C for 10″, and 60 °C for 4′). The reactions were subsequently purified with a BigDye XTerminator Purification Kit (Applied Biosystems, Waltham, MA, USA, Ref: 4376484) following the manufacturer’s instructions and sequenced via capillary electrophoresis using a SeqStudio Genetic Analyzer Cartridge (Applied Biosystems, Waltham, MA, USA, Ref: A32656) and Plate Manager software v1.1 (Applied Biosystems, Waltham, MA, USA). The_BigDye Terminator v3.1 dye set and MediumSeq run module were selected for sequencing the samples in the Plate Manager software v1.1.

### 2.9. Sequence Assembly and Clustering

The homology of the 16S rDNA sequences was checked using the Nucleotide BLASTn database (from the Nucleotide BLAST, National Center for Biotechnology Information) to compare the sequenced 16S rDNA with other 16S ribosomal RNA sequences available in the rRNA/ITS databases.

### 2.10. Mycoplasma Detection with Plasmotest^®^

The Plasmotest^®^ kit (InvivoGen, San Diego, CA, USA, Rep-pt1) allows for the specific detection of *Mycoplasma* lipopeptides, which can interact with TLR2 receptors on HEK-Blue^TM^-2 cells. The activation of the signalling cascade leads to the secretion of alkaline phosphatase, which is detected via colorimetry thanks to the HEK-BlueTM detection medium that presents a purple/blue hue in the presence of alkaline phosphatase. Cells should be used two days after passage and before reaching 80% confluency. Culture flasks for each cell line were used for Plasmotest testing. The testing protocol was performed according to the manufacturer’s recommendations, and the test results were interpreted after a 24 h incubation period at 37 °C. The kit used in the test included negative and positive controls.

### 2.11. Controls Used in the Study

The following controls were used for study validation:

- Venor^®^GeM qEP includes a positive control in the kit that was used as an additional sample. Ultrapure water included in the kit was added as a negative control.
- A *M. fermentans* DNA standard containing 1 × 10^2^ copies of strain NCTC 10117 was used as the new qPCR assay positive control. Ultrapure water was added as a negative control.
- 1/10 dilutions of the negative and positive controls included in the Plasmotest^®^ kit were used as controls and treated as any other sample.

## 3. Results

### 3.1. Mycoplasma qPCR Development

In this study, we conducted a validation test of a novel qPCR technique for the specific detection of a large number of *Mycoplasma* species. This was achieved by using 16S ribosomal RNA sequences available in rRNA/ITS databases. The PCR conditions were fine-tuned to ensure optimal detection and included the use of quantified *M. fermentans* DNA standard dilutions in triplicate. The inclusion and optimisation of an internal amplification control were thus achieved without compromising the sensitivity of the PCR reaction, thereby ensuring the efficiency of the PCR reaction.

Once the PCR conditions were established, the sensitivity of the qPCR was assessed by means of 10 CFU of lyophilised standards resuspended in 1 mL of Essential 8^TM^ DMEM/F12(Ham) (1:1) culture medium. Following isolation, 2 µL was used for qPCR, and the assays were performed in triplicate. All of the samples were detected via qPCR, and negative and positive controls validated the results that we obtained, as detailed in Table 3.

### 3.2. Comparative Analysis of Detection Methods

**Figure 2:**
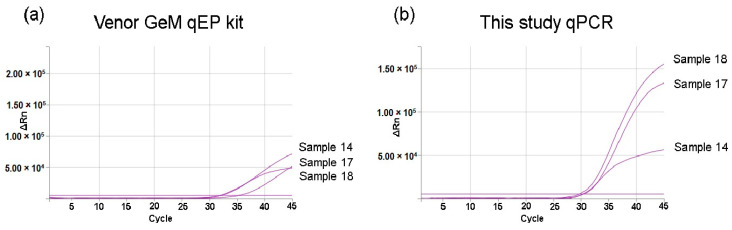
Amplification curves for Samples 14, 17, and 18 with (**a**) the Venor^®^GeM qEP kit and (**b**) this study’s qPCR. The *x*-axis indicates the number of RT-PCR cycles. The *y*-axis indicates the delta ΔRn, defined as the magnitude of the signal generated using the determined set of PCR conditions (the ΔRn value is the Rn value of an experimental reaction minus the Rn value of the baseline signal generated by the instrument).

To evaluate the new PCR technique, we performed a comparative analysis of *Mycoplasma* detection methods based on 14 different cell line supernatants of different origins at different passages (see Table 1). A total of 45 culture supernatants were used for *Mycoplasma* detection, including the new qPCR technique (being tested), a commercial Venor^®^GeM qEP kit, and a culture flask of each cell line at the same passage for Plasmotest testing. Positive results were obtained for 11 supernatants from five different cell lines (cell lines 5, 6, 7, 8, and 13). Eleven samples tested positive for *Mycoplasma* with the qPCR optimised in this study, while only 10 positive samples were detected with Venor^®^GeM qEP and Plasmotest kits. Neither the Venor^®^GeM qEP kit nor the Plasmotest kit detected one of the samples tested for cell line 5, although at different passages, the Venor^®^GeM qEP kit did not detect Sample 42, and the Plasmotest kit did not detect Sample 43 (Table 1). It is important to note that Samples 14, 17, and 18 from cell lines 5 and 6 showed 3 to 10 positive results with the Venor^®^GeM qEP kit, despite not exhibiting clear and exponential amplification, as specified in the kit’s guidelines (Figure 2).

The 11 samples that tested positive were sequenced. Their accession numbers are indicated in Table 1. Using the BLASTn database, we detected that one of the samples (Sample 15) presented 100% homology with *M. hyorhinis*. The other 10 samples showed identical sequences, and the most significant homologies (>99%) were detected with *Mycoplasma* sp. related to the *M. mycoides* cluster (*M. cottewii* and *M. yeatsii*). The complete 16S rDNA sequence was also sequenced to authenticate *Mycoplasma* identification (Accession number OP364020) [27].

## 4. Discussion

*Mycoplasma* contamination is a significant issue in cell cultures, and therefore, it is essential to have a precise, efficient, sensitive, and rapid diagnostic method for the detection of *Mycoplasma* contamination in laboratories that work with cell cultures [1]. The presence of *Mycoplasma* in cell culture alters cell growth and metabolism, producing alterations in DNA, the altered expression of surface antigens and cytokines, and causes changes in cell morphology, among many other consequences. These alterations mean that the results derived from the use of these cells are not credible or reproducible, which entails serious consequences in research and in the pharmaceutical industry [13]. As a result, most academic journals require that the research they publish certify that the study was conducted using *Mycoplasma*-free cell lines. Furthermore, the sterility of a cell line culture must be demonstrated to be deposited in a cell line bank [2,15,28].

Real-time PCR assays have gained importance in recent years for their role in detecting conserved sequences of *Mycoplasma* [1,8,10]. Due to the large number of existing *Mycoplasma* species and their genetic variability, developing a unique and infallible system for detecting *Mycoplasma* in cell culture is challenging. Although several commercial kits are available, most of them cannot be used for real-time PCR, and many of the available qPCR kits do not enjoy worldwide distribution. It is also important to consider that specific recommended protocols, such as PCR amplification programs and stated amplification sizes, are identical across different kits. These peculiarities lead us to infer that the primers used in many of these testing kits (and (potentially) the manufacturer of these kits) are the same, thus resulting in their shared strengths and weaknesses. It is also important to mention that manufacturers do not disclose what primers are used in their protocols, thereby rendering it impossible for researchers to elucidate the underlying reason(s) for discrepant test results. The documentation that accompanies the different kits includes (i) diverse sensitivity and specificity specifications and (ii) claims regarding the detection of the presence of a highly variable number of *Mycoplasma* species. Given this scenario, in combination with our experience in the detection of unclear positives with existing commercial methodologies, we have developed a new qPCR technique as an alternative *Mycoplasma* contamination detection system. The internal control that is integrated into the reaction in our detection system ensures the integrity of the qPCR and the detection of inhibitory agents. We claim that this technique is a valuable option for the scientific community and quality control laboratories for testing cell culture supernatants and resolving ambiguous or discrepant cases of *Mycoplasma* contamination.

Similar to other PCR methodologies, the new PCR methodology evaluated in this study is based on the 16S ribosomal gene of a cohort of *Mycoplasma* and *Ureoplasma* species, owing to this gene’s high degree of conservation in specific regions. Our findings regarding this new methodology corroborate its optimal sensitivity regarding the detection of the nine species that were tested, which are responsible for 98% of *Mycoplasma* contaminations, but also include atypical species related to the *M. mycoides* cluster, as previously validated through sequencing [7,27,29]. It is important to note that claims concerning the sensitivity of *Mycoplasma* detection assays are regulated by the European Pharmacopoeia, Section 2.6.7 [26]. As stated in this document, a sensitivity of 10 CFU/mL is required if a PCR assay is to be used for the detection of *Mycoplasma*. Our experiment detected a minimum of 10 CFU/mL for the nine species tested, with Cq values between 29.4 for *S. citri* and 36.74 for *M. pneumoniae* and *M. arginini*. Other PCR tests have been previously evaluated and proven to be sensitive to at least 10 CFU/mL for detecting *Mycoplasma* in culture supernatants [30], while the sensitivity of other published assays has been comparatively lower [22,31,32].

In this study, we analysed 45 supernatants from 14 cell lines at different passages. The new PCR developed in this study revealed that 11 out of these 45 supernatants tested positive for *Mycoplasma* (24.4%), although only 5 of the 14 cell lines tested positive (35.71%) positive. These positivity values are consistent with values reported in the literature [6]. Two *Mycoplasma* species were detected in the cell cultures through sequencing, namely *M. hyorninis* in one sample and a *Mycoplasma* sp. related to the *M. mycoides* cluster in 10 different samples. While *M. hyorninis* is a typical contaminant in cell cultures [7,21], the *Mycoplasma* sp. related to the *M. mycoides* cluster is usually found in infected cattle and small ruminants [33]. Previous studies have identified these microorganisms in the ear canal of goats and have reported on their presence in bovine milk and bovine lung tissue [34,35]. The contamination of cell cultures with *Mycoplasma* sp. related to the *M. mycoides* cluster has only been described twice previously [36,37]. Sequencing revealed that the species were identical in the 10 supernatants. Since they correspond to four cell lines from the same laboratory, we infer a common source of contamination, probably associated with the use of contaminated foetal bovine serum during cell line culture [36]. The complete 16S ribosomal DNA sequence cited in this study has been fully sequenced and is described in Carrillo-Avila et al. [27].

For the evaluation of the results, we have considered as true positives, those samples with a positive result based on at least two of the methodologies used. The novel PCR assay showed a congruence of 97.7% with the Venor^®^GeM qEP PCR test and the Plasmotest colorimetric test. Comparing the results of the novel PCR test with the colorimetric assay, we observed a false negative outcome for Sample 43 (cell line 5) using the Plasmotest kit. This false negative could be due to the inadequate growth of this cell culture since the confluence of this cell culture was less than optimal (i.e., <80%). The growth difficulty experienced with this cell line has been reported on by other authors [32]. Thus, determining the optimal growth conditions and confluence may pose a limitation for *Mycoplasma* detection using the colorimetric assay in the Plasmotest kit. A lack of specificity has also been described as a limitation of this colorimetric test since it can produce results for other bacteria and yeasts [38]. Nonetheless, colorimetric kits are widely accepted as an appropriate alternative tool for verifying test results [39], particularly in laboratories where routine PCR assays cannot be performed.

However, we note that 22.2% (10 out of 45) of the supernatants tested in this study indicated positivity for *Mycoplasma* using the Venor^®^GeM qEP PCR assay. This corresponds to 35.71% (5 out of 17) of the tested cell lines. The findings of this study are consistent with the results obtained using the newly developed PCR assay, except for Sample 42 (cell line 5), which tested negative with the Venor^®^GeM qEP PCR assay. Nonetheless, this sample tested positive using the new PCR test, and sequencing analysis revealed the presence of a *Mycoplasma* sp. related to the *M. mycoides* cluster that is similar to the other supernatants from cell line 5. Sample 42 also tested positive using the colorimetric assay, indicating that the negative result obtained with the Venor^®^GeM qEP PCR test was, in all probability, a false negative. This result may be due to the lower sensitivity of the Venor^®^GeM qEP PCR test with regards to *Mycoplasma* sp. related to the *M. mycoides* cluster. Notably, the amplification of *Mycoplasma* sp. in Sample 42 using the new PCR technique occurred at a higher cycle of Ct (31.87), thereby indicating a lower concentration of *Mycoplasma* in this particular sample. This decrease in sensitivity is also consistent with the non-exponential amplification observed for Samples 14, 17, and 18 from cell lines 5 and 6 using the Venor^®^GeM qEP kit, despite being considered ‘positive’ according to the kit’s specifications. Results compatible with those exposed have been previously observed by other authors, since different commercial NAT assays are frequently not capable of detecting some species of *Mycoplasma* at the <10 CFU/mL required in the European and Japanese pharmacopeias. Even some methods are not able to detect frequent *Mycoplasmas*, like *M. orale* and *M. synoviae*, and other *Mycoplasmas*, like *M. ixodetis*. These facts have led to the suggestion of the need to develop species-specific assays to supplement the deficiencies observed with commercial platforms [22].

If we take into account the premise of considering as true positives, those samples with a positive result based on at least two of the methodologies used, we can conclude a 100% sensitivity and specificity for the new PCR assay. The test is even able to detect a minimum of 10 CFU/mL for the nine species regulated by the European Pharmacopoeia, Section 2.6.7 [26]. However, an important limitation of our study is that broad-range sensitive detection cannot be guaranteed because only a limited number of known *Mycoplasma* species have been tested. It is even necessary to assay a large number of cell culture samples to have more robust sensitivity and specificity data.

## 5. Conclusions

The peculiarities of the *Mycoplasma* genus pose a significant challenge to the development of an infallible method for its detection. The occasional manifestation of inconclusive NAT outcomes and/or the requirement to perform a confirmation test (to confirm one’s test results) in conjunction with the enormous degree of heterogeneity in existing *Mycoplasma* species, the emergence of novel mutations as a consequence of natural evolution, and the ability of these species (including unusual species) to infect cell cultures indicate that the continuous evaluation and adjustment of diagnostic systems is necessary. The proposed new PCR assay for *Mycoplasma* detection was able to detect the minimum of 10 CFU/mL for the nine species regulated by the European Pharmacopoeia, Section 2.6.7 [40], in addition to showing 100% sensitivity with the 45 supernatant samples from 14 different cell lines. According to the good results obtained, the clinical significance of the new PCR assay is highest with the species and samples analysed. Despite the routine use of a commercial method for *Mycoplasma* detection, implementing an alternative *Mycoplasma* detection technique in cell culture laboratories is a step towards guaranteeing the detection of *Mycoplasma* contamination, particularly contamination by unusual species or in difficult samples that contain a low bacterium titre.

## Figures and Tables

**Table 1:** An overview of the cell lines tested, including the cell origin, passage information, and the results obtained through the present study’s qPCR, Venor^®^GeM qEP, and Plasmotest. Ct values are indicated in brackets for positives samples. The sequencing results and accession numbers are provided. Observed discrepancies are highlighted in orange.

Sample	Cell Line	Passage	Cell Origin	This Study qPCR	Venor^®^GeM qEP	PlasmoTest^®^	Sequencing Results	Accession Number
1	Cell line 1	5	Mesenchymal fat cells					
3		6						
4		7						
5		8						
6		10						
2	Cell line 2	4	Umbilical cord mesenchymal cells					
7	Cell line 3	15	Umbilical cord mesenchymal cells					
8		20						
9	Cell line 4	10	Umbilical cord mesenchymal cells					
10		12						
11		15						
12		17						
42	Cell line 5	20	iPSCs	P (29.19)	Negative	P	≈*M. mycoides* cluster	OP626186
43		21		P (31.82)	P (31.87)	Negative	≈*M. mycoides* cluster	OP626187
17		27		P (28)	P (33.02)	P	≈*M. mycoides* cluster	OP626181
13		29						
14	Cell line 6	18	iPSCs	P (27.94)	P (29.9)	P	≈*M. mycoides* cluster	OP626179
18		19		P (26.84)	P (30.61)	P	≈*M. mycoides* cluster	OP626182
44		20		P (28)	P (28.87)	P	≈*M. mycoides* cluster	OP626188
45		21		P (27.8)	P (29.51)	P	≈*M. mycoides* cluster	OP626189
15	Cell line 7	18	iPSCs	P (33.91)	P (34.48)	P	*M. hyorhinis*	OP626180
19		21						
16	Cell line 8	7	iPSCs	P (25.9)	P (26.24)	P	≈*M. mycoides* cluster	OP626183
32		12						
37		13						
40		14						
41		15						
20	Cell line 9	10	HEK-2 cells					
21	Cell line 10	23	Colon tumoral cells					
22	Cell line 11	47	hESCs					
23		49						
24		50						
25		51						
26		77						
27		79						
28	Cell line 12	23	Colon tumoral cells					
29		24						
30		25						
31		26						
33	Cell line 13	44	iPSCs					
38		46		P (28.81)	P (28.53)	P	≈*M. mycoides* cluster	OP626184
39		47		P (28.02)	P (26.66)	P	≈*M. mycoides* cluster	OP626185
34	Cell line 14	5	Skin fibroblast cells					
35		6						
36		8						

**Table 2:** Primers and probes used in the real-time PCR analysis described in this study.

Primer Name	Sequence (5′→3′)	Reference	Utility
Myco U&D-F1	CGCCTGAGTAGTACGTWCGC	[8]	PCR Forward [8]
Myco U&D-F2	TGCCTGRGTAGTACATTCGC	[8]	
Myco U&D-F3	CRCCTGAGTAGTATGCTCGC	[8]	
Myco U&D-F4	CGCCTGGGTAGTACATTCGC	[8]	
Myco-R5	GCTCGTTRCRGGACTTRACC	This study	PCR Reverse
Myco-S1 Probe	[6FAM]GGAATTGACGGGRMYCCGCAC[BHQ1]	This study	*Mycoplasma* Probe
Myco-IC-S1 Probe	[HEX]AGTAGTGTGTGCCCGTCTGT[BHQ1]	This study	Internal Control Probe

**Table 3:** Amplification Cq and Cq standard deviations for standard control detection: *A. laidlawii*, *M. arginini*, *M. fermentans*, *M. gallisepticum*, *M. hyorhinis*, *M. orale*, *M. pneumoniae*, *M. synoviae*, *S. citri*, and an isolation negative control (C-Standard). PCR negative and positive controls are included.

Sample	Cq	Cq Std. Dev.
*A. laidlawii*	36.18	±0.51
*M. arginini*	36.74	±1.34
*M. fermentans*	36.16	±0.21
*M. gallisepticum*	36.59	±0.59
*M. hyorhinis*	36.15	±0.47
*M. orale*	35.96	±0.97
*M. pneumoniae*	36.74	±1.23
*M. synoviae*	36.21	±0.07
*S. citri*	29.4	±0.21
C-Standard		
C−		
C+	30.06	±1.08

## Data Availability

The *Mycoplasma* sequences described in this study are available in the National Center for Biotechnology Information (NCBI) database under access numbers OP626179-89. The datasets generated and/or analysed during this study are available from the corresponding author on request.
